# A *Staphylococcus aureus ypfP* mutant with strongly reduced lipoteichoic acid (LTA) content: LTA governs bacterial surface properties and autolysin activity

**DOI:** 10.1111/j.1365-2958.2007.05854.x

**Published:** 2007-08

**Authors:** Iris Fedtke, Diana Mader, Thomas Kohler, Hermann Moll, Graeme Nicholson, Raja Biswas, Katja Henseler, Friedrich Götz, Ulrich Zähringer, Andreas Peschel

**Affiliations:** 1 Cellular and Molecular Microbiology Division, University of Tübingen, Department of Medical Microbiology and Hygiene 72076 Tübingen, Germany; 2 Immunochemistry and Biochemical Microbiology, Research Center Borstel-Center for Medicine and Biosciences 23845 Borstel, Germany; 3 Organic Chemistry, University of Tübingen 72076 Tübingen, Germany; 4 Microbial Genetics, University of Tübingen 72076 Tübingen, Germany

## Abstract

Many Gram-positive bacteria produce lipoteichoic acid (LTA) polymers whose physiological roles have remained a matter of debate because of the lack of LTA-deficient mutants. The *ypfP* gene responsible for biosynthesis of a glycolipid found in LTA was deleted in *Staphylococcus aureus* SA113, causing 87% reduction of the LTA content. Mass spectrometry and nuclear magnetic resonance spectroscopy revealed that the mutant LTA contained a diacylglycerol anchor instead of the glycolipid, whereas the remaining part was similar to the wild-type polymer except that it was shorter. The LTA mutant strain revealed no major changes in patterns of cell wall proteins or autolytic enzymes compared with the parental strain indicating that LTA may be less important in *S. aureus* protein attachment than previously thought. However, the autolytic activity of the mutant was strongly reduced demonstrating a role of LTA in controlling autolysin activity. Moreover, the hydrophobicity of the LTA mutant was altered and its ability to form biofilms on plastic was completely abrogated indicating a profound impact of LTA on physicochemical properties of bacterial surfaces. We propose to consider LTA and its biosynthetic enzymes as targets for new antibiofilm strategies.

## Introduction

The bacterial cell wall is vital for containing the high osmotic pressure of the cytoplasm, determining the cell shape and protecting the cell against harmful factors from the environment. In addition to a thick fabric of peptidoglycan the Gram-positive cell envelopes usually contain polymers composed of sugars or sugar alcohols modified with additional sugars, amino acids or choline. While the species of the high G+C branch of Gram-positive bacteria often contain lipoglycans without phosphate in the backbone (Sutcliffe and Russell, 1995) those from the low G+C branch produce polymers with phosphodiester bond-connected repeating units called teichoic acids (TAs), which can be classified in two subtypes. The wall TA (WTA) is covalently linked to the peptidoglycan while the lipo-TA (LTA) is anchored in the outer leaflet of the cytoplasmic membrane via a glycolipid (Neuhaus and Baddiley, 2003). Most TA-producing bacteria have both types of molecules, which are usually different in structure, biosynthesis and presumed functions. LTA and WTA both extend to the bacterial cell surface and are assumed to influence surface properties.

While it has been possible to generate WTA-deficient mutants (Lazarevic *et al*., 2002; Weidenmaier *et al*., 2004; D'Elia *et al*., 2006) LTA is an essential molecule for viability in *Staphylococcus aureus* (Gründling and Schneewind, 2007a). TA structures and biological activities have been studied to some extent in *S. aureus* as WTA and LTA seem to contribute to the virulence potential of this major human pathogen (Morath *et al*., 2001; Weidenmaier *et al*., 2004). Many functions have been attributed to LTA, e.g. influence on cell division (Gründling and Schneewind, 2007a), modulation of autolytic enzyme activities (Bierbaum and Sahl, 1987), maintenance of cation homeostasis (Hughes *et al*., 1973), resistance to cationic antimicrobial molecules (Peschel *et al*., 1999), impact on biofilm formation (Gross *et al*., 2001; Fabretti *et al*., 2006) and pro-inflammatory properties (Morath *et al*., 2001), reviewed in Neuhaus and Baddiley (2003). However, many of these studies are based on mutants with structural alterations in both LTA and WTA and do not allow to define LTA-specific effects.

Lipoteichoic acid biosynthesis and sequence of assembly are only incompletely understood. LTA is composed of poly(glycerolphosphate) ([Gro-*P*]_n_) attached to the glycolipid anchor β-d-Glc*p*^II^-(1→6)-β-d-Glc*p*^I^-(1→3)-diacylglycerol (DGlcDAG) in staphylococci and many bacilli (Fischer, 1988). The YpfP protein, a glycolipid synthase, has been shown to mediate DGlcDAG synthesis in *Bacillus subtilis* (Jorasch *et al*., 1998) and *S. aureus* (Jorasch *et al*. 2000). However, a *S. aureus* RN4220 *ypfP::cat* mutant still produced LTA, even at increased amounts, and with changes in the chemical composition (Kiriukhin *et al*., 2001). Recently, the gene located downstream of *ypfP*, named *ltaA*, has been shown to be involved in LTA biosynthesis, probably by translocating DGlcDAG from the inner to the outer leaflet of the cytoplasmic membrane (Gründling and Schneewind, 2007b). The LTA polymer is synthesized by the LtaS protein (Gründling and Schneewind, 2007a).

In an attempt to better understand LTA biosynthesis and function, we generated a *ypfP* deletion mutant in *S. aureus* SA113 and confirmed that the mutant still produces LTA although with the polymer attached to diacylglycerol (DAG) instead of DGlcDAG. The LTA content of this mutant was 87% reduced compared with the wild type, which indicates a profound difference to the above mentioned RN4220 mutant. The SA113 mutant enabled us to study the impact of reduced LTA content on relevant cell wall properties. We demonstrate a role of LTA in regulation of autolysin activity, surface hydrophobicity and biofilm formation, while other key functions such as surface protein patterns, growth kinetics and WTA content were not or hardly affected. This study represents a basis for understanding the role of LTA in bacterial physiology and sheds new light on the pathway of LTA biosynthesis.

## Results

### Deletion of the DGlcDAG synthase gene *ypfP* in *S. aureus* SA113

**Fig. 1:**
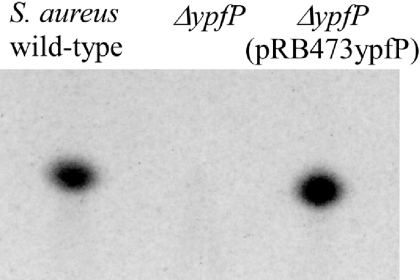
Detection of *S. aureus* SA113 glycolipids by TLC. Glycolipids were visualized on TLC plates with α-naphthol. The spot missing in the *S. aureus ypfP* mutant was identified as DGlcDAG.

The *ypfP* gene previously shown to be essential for DGlcDAG biosynthesis (Kiriukhin *et al*., 2001) was inactivated in *S. aureus* SA113 by gene replacement. Polar lipids of the resulting mutant were analysed by thin-layer chromatography (TLC) and found to lack a lipid spot present in the wild type. The lipid in question was stainable with α-naphthol, which is indicative of glycolipids (Fig. 1). Complementation of the mutant with a wild-type copy of the deleted gene restored glycolipid production (Fig. 1). The lipid lacking in the mutant was analysed by gas chromatography coupled to mass spectrometry (GC-MS) and found to contain glucose, glycerol (Gro) and fatty acids (predominantly branched C15 acyl chains; data not shown), which is in accordance with the expected constituents of DGlcDAG.

### *ypfP* deletion causes strongly reduced LTA content but unaltered WTA content in *S. aureus* SA113

**Fig. 2:**
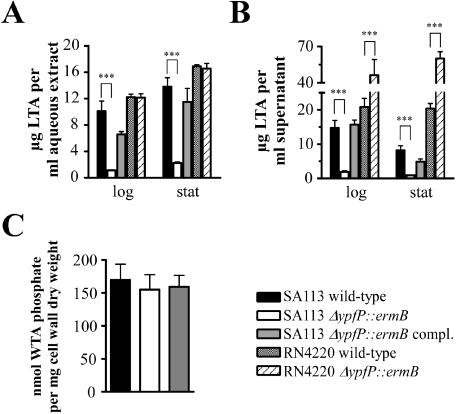
Quantification of teichoic acids. Cell-bound LTA (A) or LTA in the supernatant (B) of *S. aureus* strains grown to logarithmic phase (log) or overnight (stat) was quantified by ELISA. The concentration of LTA in cell lysates or supernatants was normalized to cultures with an OD_578_ of 1. Organic phosphate was determined as a measure for WTA concentration (C). Data represent the means ± SEM of at least three independent experiments (***P < 0.001, unpaired, two-tailed *t*-test).

As free DGlcDAG has been considered as the acceptor molecule for polymerization of the LTA polymer (Fischer, 1997), DGlcDAG-deficient mutants may be expected to have a defect in LTA biosynthesis. Therefore, we quantified the LTA amounts in crude cell extracts and culture supernatants of wild-type and *ypfP* mutant bacteria from logarithmic and stationary growth phases by enzyme-linked immunosorbent assay (ELISA) using a monoclonal antibody that detected the [Gro-*P*]_n_ backbone of wild-type and mutant LTA equally well (see *Experimental procedures*). The amounts of LTA in logarithmically growing mutant bacteria (Fig. 2A) and corresponding culture supernatants (Fig. 2B) were strongly reduced to 11% and 13%, respectively, of wild-type levels. Similar differences were observed with bacteria grown to the stationary phase (16% and 11% reduced LTA contents in mutant bacteria and culture supernatants respectively). LTA amounts were close to normal in the complemented mutant strain. Wild-type and *ypfP* mutant strains had very similar amounts of WTA (Fig. 2C) indicating that reduced LTA is not compensated by increased WTA production.

These data are contradictory to those from the recently described *S. aureus* RN4220 *ypfP::cat* mutant, which has been shown to produce two- to threefold higher amounts of LTA compared with the parental strain (Kiriukhin *et al*., 2001). Our *ypfP* mutant exhibited further profound differences in several aspects of bacterial physiology compared with the RN4220 mutant and seems to harbour a different perturbation of LTA biosynthesis (see below). In order to study whether this differences result from peculiarities in mutant construction or from differences in the strain background, our *ypfP* mutation was transferred from *S. aureus* SA113 to *S. aureus* RN4220 by phage transduction. Interestingly, the resulting RN4220 Δ*ypfP::ermB* mutant did not reveal a reduced content of LTA. Instead, the amount of cell-associated LTA of the mutant was equal to that of the parental strain in both, logarithmic and stationary growth phase (Fig. 2A) and culture supernatants contained two- to threefold increased LTA concentrations (Fig. 2B). This behaviour is reminiscent of that of the recently described RN4220 mutant with a disrupted *ypfP* gene suggesting that the consequences of a *ypfP* mutation is dependent on the corresponding strain background. In order to confirm this assumption, we generated two new SA113 *ypfP* mutants (i) by transducing the Δ*ypfP::ermB* mutation from RN4220 back to SA113 and (ii) by targeted gene replacement using another antibiotic resistance cassette [spectinomycin (*spc*) instead of erythromycin (*ermB*)]. Both new SA113 *ypfP* mutants behaved again like our first mutant with strongly reduced cell-associated or released LTA contents compared with wild type (data not shown) thereby confirming that the strain backgrounds, SA113 versus RN4220, are decisive for the resulting LTA phenotype for so far unknown reasons.

### Compositional analyses of the LTA purified from parental and *ypfP* mutant strains

In order to study possible structural differences in LTA from *S. aureus* SA113 wild-type and SA113 *ypfP* mutant strains, LTA was isolated from both strains according to established protocols by extraction of cell lysates with butanol and purification by hydrophobic interaction chromatography (HIC) using octylsepharose columns (Morath *et al*., 2001). Because of its low abundance LTA from the mutant was obtained only at very low yields.

**Fig. 3:**
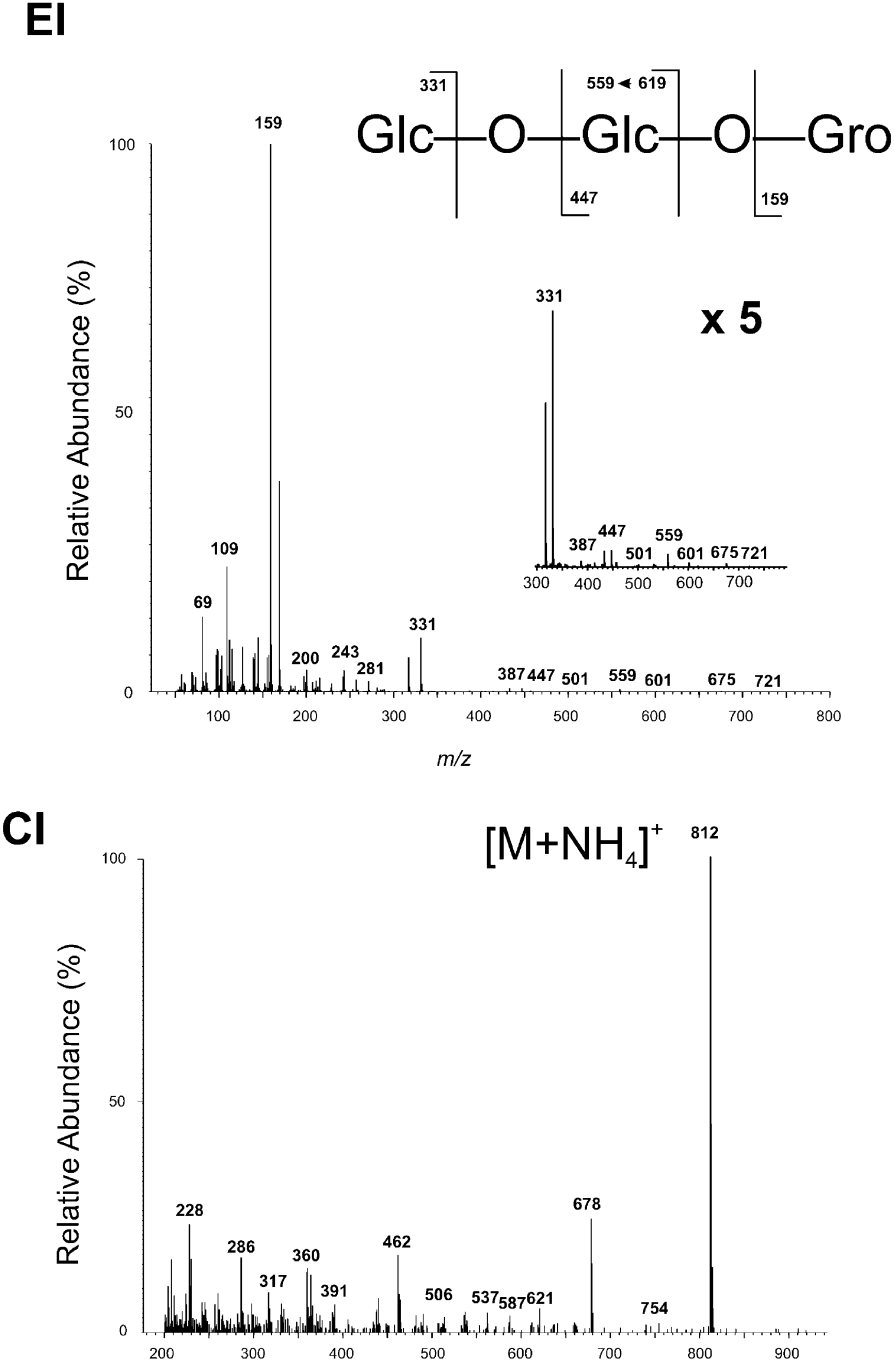
GLC-MS spectra of the per-O-acetylated glycan isolated from *S. aureus* SA113 wild-type LTA. The EI mass spectrum (top) shows the predominant *m/z* = 159 fragment for the Gro unit. Other characteristic fragments originating from the terminal hexose in the glycerol gentiobiose pseudotrisaccharide are shown with enlarged intensity (5×) on the right. The base peak (*m/z* = 812) in the CI MS spectrum (bottom) of the per-O-acetylated glycan represents the pseudomolecular ion [M+NH_4_]^+^ of the per-O-acetylated glycerol β-gentiobiose pseudotrisaccharide (Glc-O-Glc-O-Gro).

Gas-liquid chromatography (GLC)-MS analyses of the fatty acids in the LTA of both strains revealed an identical fatty acid profile (*iso*-15:0 and *iso*-17:0) whereby *iso*-15:0 dominated. Methanolysis followed by per-O-acetylation and subsequent GLC-MS revealed the expected components of the repeating glycerophosphate in both LTAs, whereby unsubstituted Gro, Gro phosphate di-methylester (Gro-*P*), alanylated Gro (Gro-d-Ala) and 2-acetamido-2-deoxy-α-d-glucopyranosyl-substituted Gro (Gro-GlcNAc) were found in comparable amounts and proportions in LTA preparations from parental and mutant strains. However, only in case of the wild-type strain, a late eluting peak (46.46 min) could be detected in the GLC-MS (data not shown) suggesting that this compound, maybe a short oligosaccharide, is not present in the mutant. The electron impact (EI) mass spectrum (Fig. 3) showed diagnostic fragment ions at *m/z* = 331 for a terminal peracetylated hexosyl (Glc^II^) and *m/z* = 159 assigned to the Gro residue. Chemical ionization (CI) with ammonia revealed a pseudomolecular ion ([M + NH_4_]^+^*m/z* = 812), which was compatible with the per-O-acetylated glyceryl β-gentiobioside (Glc-O-Glc-O-Gro) (Fig. 3).

### Nuclear magnetic resonance analyses of the glyceryl β-gentiobioside (lipid anchor backbone)

**Fig. 4:**
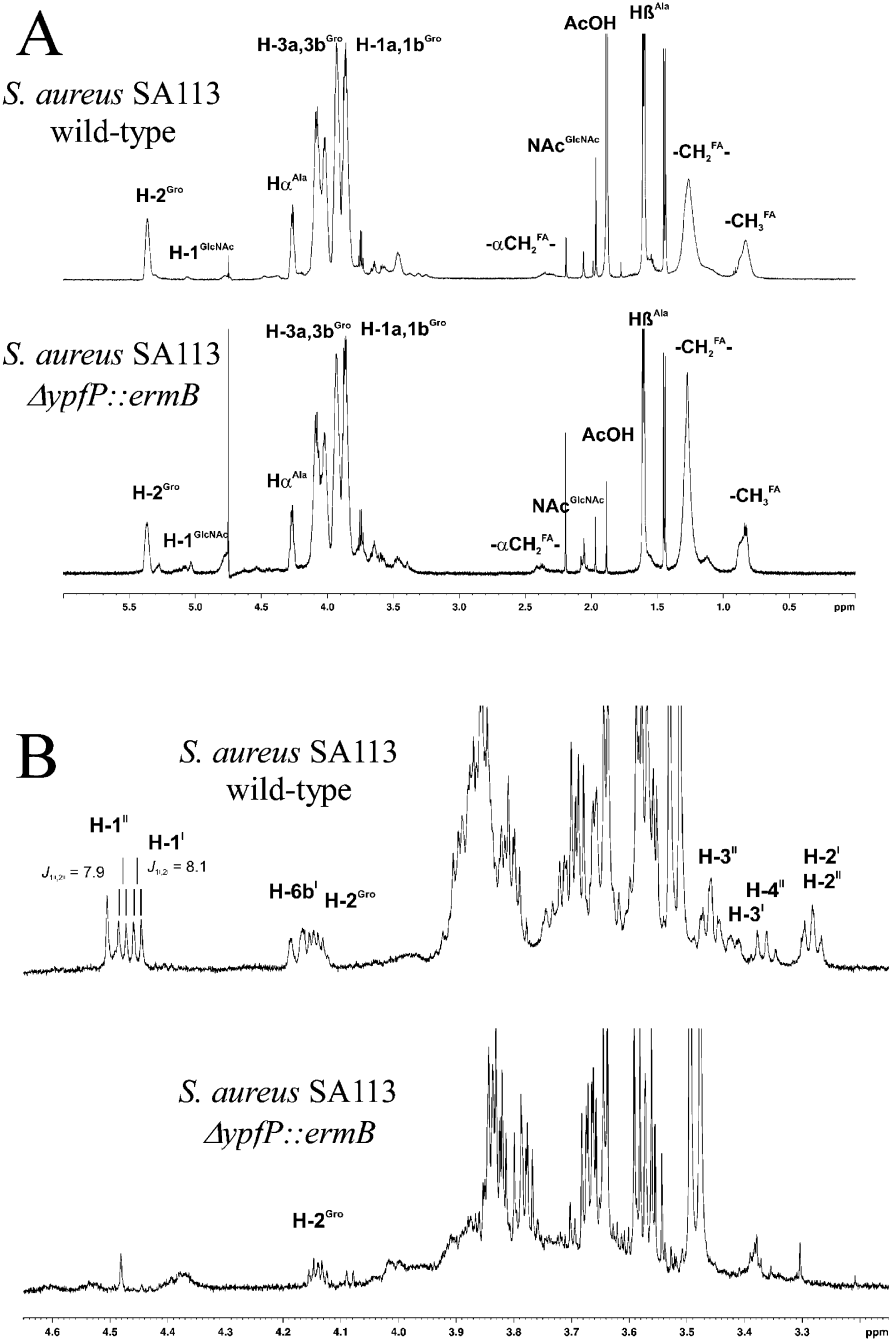
NMR analyses. A. ^1^H NMR spectra (600 MHz, D_2_O, 300 K) of HIC-purified intact LTA. Characteristic resonances for the [Gro-*P*]_n_ repeating units, fatty acids and Gro-*P* substituents (d-Ala and α-d-GlcNAc) are indicated. B. ^1^H NMR spectra (600 MHz, D_2_O, 300 K) of the putative glycan backbone fragments isolated from HIC-purified LTA after HF treatment, de-O-acylation and GPC. Signals indicated in the glycerol β-gentiobiose pseudotrisaccharide (top) lack in the sample from the *S. aureus* SA113 Δ*ypfP::ermB* mutant (bottom). Resonances in the region of 3.4–3.9 p.p.m., which appear in both preparations, arise from incompletely degraded LTA repeating units [(Gro-*P*)_n_ (Gro-*P*-Ala)_n_ and (Gro-*P*-GlcNAc)_n_ with n ∼ 1–4] co-eluting in GPC with the glycerol β-gentiobioside.

^1^H nuclear magnetic resonance (NMR) analyses of underivatized and intact LTA preparations from *S. aureus* SA113 wild-type and *ypfP* mutant strains were carried out in water (D_2_O) (Fig. 4A). Diagnostic ^1^H NMR signals from substituted H-2^Gro^ (5.45 p.p.m.) of the [Gro-*P*]_n_ repeating unit, as well as resonances assigned to H-1a,b^Gro^, H-3a,b^Gro^ (3.7–4.2 p.p.m.), and fatty acids (1.2–0.8 p.p.m.) dominated in the ^1^H NMR spectrum, which was in very good agreement with data published elsewhere (Morath *et al*., 2001). In addition to signals from the [Gro-*P*]_n_ repeating units, resonances assigned to the Gro-*P* substituents (d-Ala and α-GlcNAc) could also be identified in comparable intensities in both LTA preparations. Our results from the ^1^H NMR analyses of the intact LTA suggest that both LTA preparations are similar with respect to the structure of the repeating units of the [Gro-*P*]_n_ as well as to their degree of substitution with d-Ala and α-GlcNAc respectively. Following a previously described ^1^H NMR approach (Morath *et al*., 2001), we identified a chain length of n ∼ 18 for the wild-type SA113 and only n ∼ 12 for the *ypfP* mutant. However, signals of the glycerol β-gentiobiose lipid backbone anchor could not be identified by this method. This is mainly due to limited resolution of the ^1^H NMR signals of the LTA in water (D_2_O), in which it is present in micelle-forming aggregates leading to badly resolved proton signals. Moreover, due to the high polymerization degree of the [Gro-*P*]_n_ repeating units the glyceryl β-gentiobiose moiety represents only ∼3% of the total molecular mass in the intact LTA molecule. This explains why the glycan backbone in the underivatized LTA cannot be analysed by ^1^H NMR spectroscopy. Therefore, we decided to isolate the target glycan backbone structure of the lipid anchor of LTA of both strains aiming to determine their structure(s) separately and in more detail.

To this end, purified LTA from *S. aureus* SA113 wild type and the *ypfP* mutant was HF-treated to remove the [Gro-*P*]_n_ repeating units and de-O-acylated to eliminate fatty acids (*iso*-15:0 and *iso*-17:0) from the lipid anchor. The remaining glycan backbone was further purified by gel permeation chromatography (GPC) (Sephadex G10). GPC did not reveal highly purified glycan as the preparations still contained components from incompletely degraded LTA (Gro-d-Ala, Gro-GlcNAc and [Gro-*P*]_n_ with n ∼ 1–4). However, careful ^1^H NMR analyses using two-dimensional (2D) techniques (Correlation Spectroscop Y, COSY, Total Correlated Spectroscop Y, TOCSY) allowed the unequivocal determination of the target structure (Fig. 4B). As indicated in Table S1, the glycan backbone of the wild-type strain showed *inter alia* signals, which were diagnostic for the β-interlinked β-gentiobioside in pseudotrisaccharide β-d-Glc*p*^II^-(1→6)-β-d-Glc*p*^I^-(1→3)-Gro (H-1^II^, 4.483 p.p.m., *J*_1,2_ = 7.9 Hz; H-1^I^, 4.457 p.p.m., *J*_1,2_ = 8.1 Hz). The rest of the signals were found to be in excellent agreement with ^1^H NMR data published elsewhere (Thompson *et al*., 2002; Stadelmaier *et al*., 2006) (Table S1).

Taken together, these findings demonstrate that the LTA of the SA113 *ypfP* mutant lacks the gentiobiose disaccharide and bears a shortened [Gro-*P*]_n_ chain attached directly to the DAG lipid moiety. On the other hand, the substitution pattern of the d-Ala and α-GlcNAc residues attached to the C-2 position of the [Gro-*P*]_n_ were found not to be altered in the mutant LTA.

### Eighty-seven per cent reduced LTA content has no major impact on *in vitro* growth, salt tolerance and antimicrobial peptide susceptibility but leads to reduced viability at late stationary phase

**Fig. 5:**
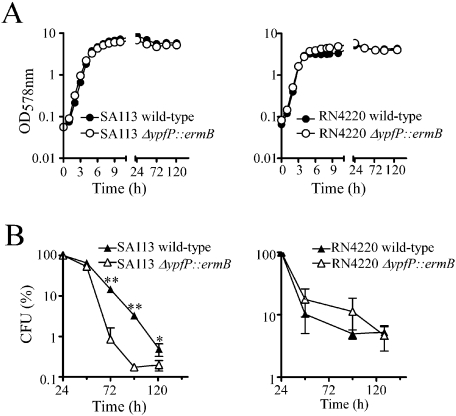
Growth (A) and stationary-phase survival (B) curves of *S. aureus* strains. The numbers of live bacteria in cultures grown for 24 h were defined as 100% in (B). Data represent the means ± SD of three independent cultures (*P < 0.05; **P < 0.01 versus wild type; unpaired, two-tailed *t*-test).

The *S. aureus* SA113 *ypfP* mutant represents the first stable bacterial strain with strongly reduced LTA content thereby providing a means to study the role of LTA in basic bacterial processes. *In vitro* growth and survival behaviours of parental and mutant strains were compared. Surprisingly, growth rates of wild-type and mutant strain were indistinguishable (Fig. 5A) indicating that *S. aureus in vitro* growth is not dependent on normal LTA amounts. However, the SA113 *ypfP* mutant had a more than 10-fold reduced capacity to survive at late stationary phase after several days of incubation (Fig. 5B) while the corresponding RN4220 mutant, which lacks the glycolipid but contains normal amounts of LTA, did not show such a survival defect (Fig. 5). Therefore, the reduced ability of the SA113 *ypfP* mutant to cope with starvation seems to result from LTA depletion rather than lack of the glycolipid. In contrast to the *S. aureus* RN4220 *ypfP::cat* mutant (Kiriukhin *et al*., 2001), none of our Δ*ypfP* mutants showed defects in growth on Baird-Parker agar (data not shown).

**Fig. 6:**
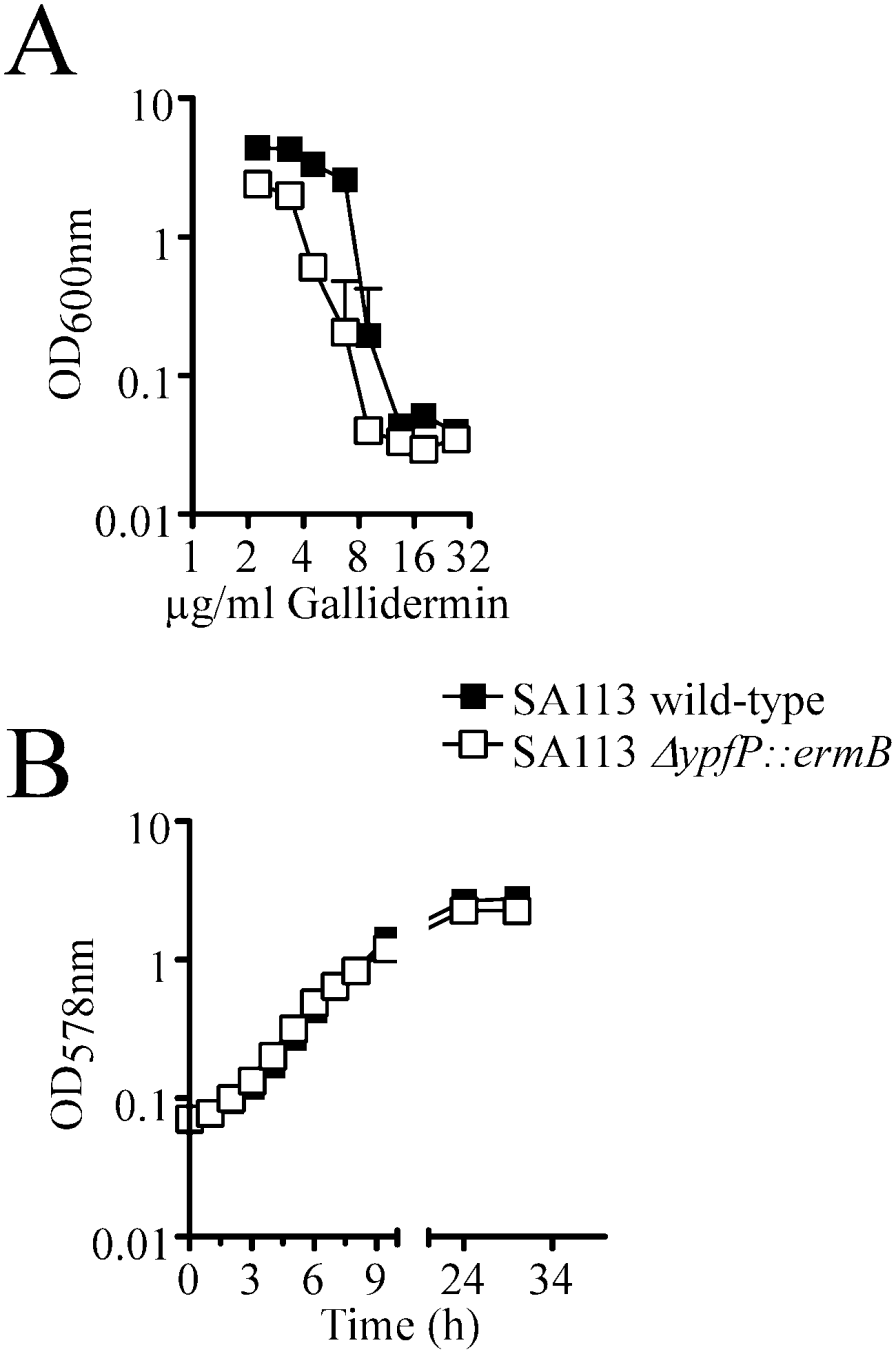
Impact of *ypfP* deletion on growth under unfavourable conditions. A. Growth for 6 h in the presence of increasing concentrations of gallidermin. B. Growth in the presence 2.5 M NaCl. Data represent the means ± SD of three independent experiments.

Staphylococci are exposed to considerable amounts of cationic antimicrobial peptides (CAMPs) in epithelial secretions produced by host cells (defensins) or other bacteria (bacteriocins) in their natural habitats (Peschel and Collins, 2001; Peschel and Sahl, 2006). As teichoic acid structure, in particular the d-Ala modifications, are important determinants for bacterial CAMP susceptibility (Peschel *et al*., 1999; Collins *et al*., 2002) we compared the minimal inhibitory concentrations (MICs) of the staphylococcal bacteriocin gallidermin and the defensin-like CAMP tachyplesin 1 towards SA113 parental and *ypfP* mutant strains. Susceptibilities of wild type and mutant were similar with gallidermin (9 μg ml^−1^ and 7 μg ml^−1^ respectively; Fig. 6A) or tachyplesin 1 (12 μg ml^−1^ and 13 μg ml^−1^ respectively) indicating that the content of LTA *per se* has no major impact on CAMP susceptibility. MICs of the glycopeptide antibiotic vancomycin were also similar for the SA113 wild-type and mutant strains (4 and 3 μg ml^−1^ respectively).

*Staphylococcus aureus* is able to tolerate very high salt concentrations on human skin. As LTA has been implicated in controlling the ionic milieu in the cell envelope (Neuhaus and Baddiley, 2003), we address the impact of LTA on halotolerance. However, SA113 wild-type and *ypfP* mutant strains grew equally well in the presence of 2.5 M NaCl (Fig. 6B) indicating that LTA depletion does not affect *S. aureus* salt tolerance.

### Deletion of *ypfP* has no major impact on surface protein and autolysin patterns but affects autolysin activity

**Fig. 7:**
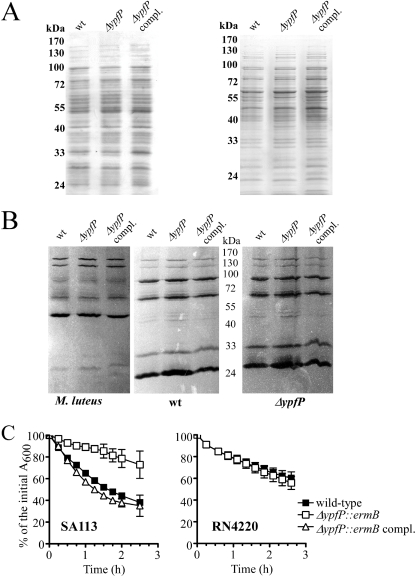
Impact of *ypfP* deletion on cell wall proteins. A. Surface protein patterns of cells grown to logarithmic (left) or stationary (right) phase. SA113 wild-type and SA113 Δ*ypfP::ermB* mutant strains did not differ in overall amounts of cell wall proteins. Ten micrograms of surface proteins of the indicated SA113 strains were separated in 10% polyacrylamide gels and stained with Coomassie brilliant blue R-250. B. Zymographic analyses. Surface proteins (20 μg) of SA113 log-phase bacteria were separated in 10% SDS-polyacrylamide gels containing heat-killed *Micrococcus luteus* (*M. luteus*), *S. aureus* SA113 wild type (wt) or *S. aureus* SA113 Δ*ypfP::ermB* mutant cells (Δ*ypfP*). Active bacteriolytic enzymes appear as clear zones in the opaque gels. The inverse pictures are shown. C. Spontaneous bacterial autolysis. Data represent the means ± SD of three independent experiments.

Lipoteichoic acid has been assumed to serve as a scaffold structure for anchoring surface proteins non-covalently to the cell wall (Navarre and Schneewind, 1999; Neuhaus and Baddiley, 2003). This hypothesis prompted us to compare the overall patterns of SA113 wild-type and mutant cell wall-associated proteins. Surface proteins were isolated by gentle treatment of osmotically stabilized bacterial cells with the cell wall-degrading enzyme lysostaphin and subjected to sodium dodecyl sulphate polyacrylamide gel electrophoresis (SDS-PAGE). Only minor changes were detected while the major protein bands were unaffected, irrespective of whether proteins from logarithmic or stationary growth phases were compared (Fig. 7A).

Lipoteichoic acid has been particularly implicated in binding of murein-hydrolytic enzymes (autolysins), required for cell wall turn-over and cell division (Bierbaum and Sahl, 1987). In order to study possible differences in autolysin amounts of *S. aureus* SA113 wild type and *ypfP* mutant, cell wall-associated proteins from log-phase bacteria were separated by SDS-PAGE and autolysins were subsequently visualized as clearing zones after washing out SDS from polyacrylamide gels to which heat-inactivated bacterial cells had been added. Again, these ‘zymograms’ did not reveal any differences between wild-type and *ypfP* mutant, irrespective of whether *Micrococcus luteus*, *S. aureus* SA113 wild-type or *ypfP* mutant had been added to the gels as autolysin substrates (Fig. 7B).

The autolytic behaviours of SA113 wild type and *ypfP* mutant were also compared upon artificial activation of autolysins by washing intact bacterial cells in ultrapure water. Autolysis was then monitored in low-ionic strength buffer containing Triton X-100 to enhance autolytic activities. Interestingly, the SA113 *ypfP* mutant revealed a considerable reduction in autolysis rate compared with wild type and complemented mutant (Fig. 7C) confirming the notions that LTA is critical in controlling autolysin activity (Bierbaum and Sahl, 1987; Smith *et al*., 2000). However, this reduced activity does not appear to result from reduced amounts of autolytic enzymes in the cell wall but from lower enzymatic activities. In accord with this notion, the RN4220 strain pair revealed no such differences (Fig. 7C).

### LTA governs bacterial surface hydrophobicity and has a crucial impact on biofilm formation

**Fig. 8:**
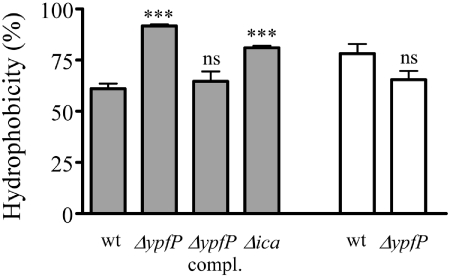
Cell surface hydrophobicity of *S. aureus* strains. The propensities of *S. aureus* wild type (wt), Δ*ypfP::ermB* (Δ*ypfP*), Δ*ypfP::ermB* (pRB473ypfP) (Δ*ypfP* compl.) and Δ*ica* (Δ*ica*) to associate with the organic or aqueous phase in a dodecane/buffer mixture were determined. SA113 strains, grey bars; RN4220 strains, white bars. Data represent the means ± SEM of at least nine counts from three independent experiments (ns, not significant, ***P < 0.0001 versus wild type; unpaired, two-tailed *t*-test).

Lipoteichoic acid molecules are exposed on the bacterial surface and the high number of charged phosphate and d-Ala residues has probably a considerable impact on the bacterial surface structure. Such physicochemical properties are critical in staphylococcal adherence to artificial surfaces such as catheters or heart valve prostheses and in resulting biofilm-associated infections (Davey and O'Toole, 2000; Götz, 2002). In order to study possible differences in overall surface hydrophobicity SA113 wild-type and *ypfP* mutant strains were compared for their propensity to associate with aqueous or organic phase after vigorous shaking in a mixture of phosphate buffer and dodecane. As shown in Fig. 8, the *ypfP* mutant revealed a significant increase in hydrophobicity while the complemented mutant behaved like the wild type. In contrast, RN4220 wild type and *ypfP* mutant exhibited the same hydrophobicity (Fig. 8) indicating that LTA depletion but not lack of the glycolipid leads to increased hydrophobicity. The previously described SA113 *ica* mutant (Cramton *et al*., 1999), which does not produce the slime polymer polysaccharide intercellular adhesin (PIA) needed for cell-to-cell binding within biofilms (Heilmann *et al*., 1996a), also revealed reduced hydrophobicity although to a lower extent than the SA113 *ypfP* mutant (Fig. 8).

**Fig. 9:**
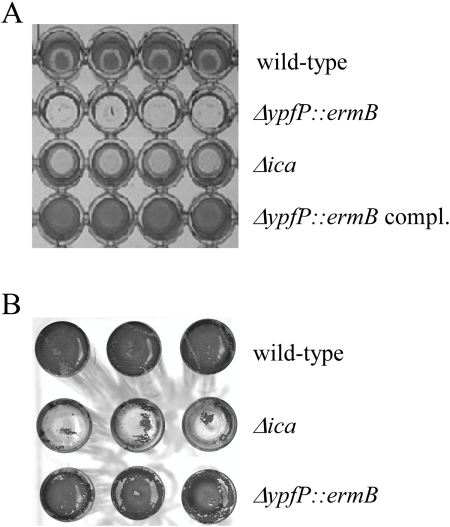
*In vitro* biofilm formation. A. *S. aureus* SA113 strains adhering to polystyrene microtitre plates were stained with safranin. Four replica are shown. B. Biofilm formation as in (A) but in glass tubes. Three replica are shown.

SA113 strains were also analysed for their capacity to form biofilms during growth in polystyrene microtitre plates (Fig. 9A) or in glass tubes (Fig. 9B). While the wild type clearly formed biofilms on polystyrene the *ypfP* mutant seemed not to adhere and had completely disappeared from the wells after washing. Biofilm formation was even stronger reduced with the *ypfP* mutant than with the PIA-deficient *ica* mutant (Cramton *et al*., 1999). Wild-type phenotype was restored in the complemented mutant. In glass tubes, only the *ica* mutant exhibited a defect in biofilm formation. However, the *ypfP* mutant formed a biofilm on glass confirming the notion that changes in hydrophobicity of either bacteria or biomaterials are crucial for the ability to form biofilms although the exact nature of molecular interactions remain elusive. As the RN4220 wild type showed hardly any biofilm formation the RN4220 was omitted from this experiment. Taken together, our study indicates that the LTA content governs the physicochemical surface properties of *S. aureus* and enables biofilm formation.

## Discussion

The presence of LTA or of related lipoglycans in the cell walls of most Gram-positive bacteria and the fact that bacterial mutants lacking LTA or with reduced LTA content have never been described have led to the assumption that LTA plays an important and indispensable role in the Gram-positive cell wall. This notion has recently been confirmed by inducible inactivation of *ltaS* in *S. aureus* (Gründling and Schneewind, 2007a). While several possible functions have been assigned to LTA clear and reliable data confirming such assumptions have hardly been available. This study describes for the first time a stable mutant with strongly reduced LTA content, which offers new opportunities for investigating the role of LTA. As our SA113 Δ*ypfP* mutant is growing normally under laboratory conditions and exhibit no obvious signs of cellular dysfunctions, at least in growing cells, we conclude that the residual 13% of LTA in the mutant are sufficient to fulfil most of the LTA functions in the *S. aureus* cell envelope. However, viability of the SA113 Δ*ypfP* mutant was abrogated at late stationary phase indicating that the mutation affects bacterial functions under suboptimal conditions. This defect can be attributed to the depletion of LTA rather than the lack of the glycolipid as our RN4220 Δ*ypfP* mutant with normal cellular LTA content did not differ from its parental strain in terms of stationary-phase survival.

Amazingly, RN4220 *ypfP* mutants exhibit quite different phenotypes than SA113 *ypfP* mutants apart from the absence of DGlcDAG. While SA113 *ypfP* mutants had strongly reduced LTA contents the RN4220 *ypfP* mutant produced equal cellular amounts of LTA and even two to three times more LTA in supernatants compared with the parental strain. Moreover, spontaneous autolysis and surface hydrophobicity were altered in SA113 but not in RN4220 *ypfP* mutants. The different consequences of *ypfP* deletion were clearly dependent on the strain background as several independently generated *ypfP* mutants in SA113 (this study) and in RN4220 (one in this study, one previously described by the Neuhaus lab) each revealed consistent, strain-specific phenotypes. It might be speculated that subtle, yet unknown differences in the LTA polymerization machineries determine the impact of the lack of the glycolipid on LTA biosynthesis rates. Major differences in basic cellular functions are not unusual in *S. aureus* and have been described, for example, for arginine catabolism (Diep *et al*., 2006), sigma B-dependent regulation (Gertz *et al*., 1999) or WTA composition (Endl *et al*., 1983).

Nuclear magnetic resonance analyses revealed that the gentiobiose disaccharide is lacking in the LTA of the SA113 Δ*ypfP::ermB* mutant. Instead, the mutant LTA polymer is directly attached to DAG and is considerably shorter compared with wild-type LTA. This finding is in part congruent with preliminary structural analyses (Kiriukhin *et al*., 2001) and with altered electrophoretic migration (Gründling and Schneewind, 2006) of LTA from the RN4220 *ypfP* mutant. It is obvious that the glycolipid is not an essential acceptor molecule for the LTA polymer, a notion that is further supported by the presence of LTA molecules with the polymer attached to DAG instead of DGlcDAG in certain *Bacillus* species (Iwasaki *et al*., 1986; Fischer, 1988). DAG may either represent the primary acceptor for the LTA polymer, replaced at later stages of the biosynthetic pathway by DGlcDAG or the LTA polymerase may use DAG as acceptor with low efficiency. Investigating which of these alternative hypotheses holds true will require a detailed characterization of the biosynthetic machinery.

The SA113 *ypfP* mutant made it possible to analyse for the first time the impact of 87% reduced LTA content on *S. aureus* cell wall protein attachment, autolysin activity and physicochemical surface properties. While several proteins are covalently attached to the staphylococcal cell wall (Foster and Hook, 1998; Navarre and Schneewind, 1999; Clarke and Foster, 2006), many surface proteins are retained in the cell envelope via non-covalent interactions (Chavakis *et al*., 2005). As LTA represents an anchoring scaffold for non-covalently attached surface proteins in *Listeria monocytogenes* (Cabanes *et al*., 2002) and *Streptococcus pneumoniae* (Bergmann and Hammerschmidt, 2006) we expected a profound change in *S. aureus* cell wall protein patterns in the absence of most of the LTA molecules. However, the surface protein patterns revealed only minor changes indicating either that the vast majority of these proteins are attached independently of LTA or that the residual LTA amounts in the SA113 *ypfP* mutant are sufficient to achieve normal protein allocation. There were also no considerable changes in autolysin patterns detectable in zymograms. These data add to a recent study on autolysin attachment and activity in a *S. aureus dltA* mutant (Peschel *et al*., 2000), which lacks the positively charged d-Ala groups. As *dltA* mutant cell walls contained reduced amounts of autolysins one might propose that the overall net charge in the cell envelope may have a more important role in protein attachment than the mere presence of LTA.

While autolysin amounts and patterns were largely unaffected in the SA113 *ypfP* mutant, autolysin activity was clearly reduced suggesting that LTA affects the activity or substrate accessibility of autolytic enzymes. We can only speculate how LTA may impact on autolysins. While the autolysins may be directly bound to peptidoglycan the charged phosphate and d-Ala groups of LTA may affect autolysin conformation or the availability of LTA-bound magnesium or calcium ions may play a critical role in the enzymatic activity. Accordingly, the activity of autolysins has been shown to be stimulated by bivalent cations at low ionic strength (Bierbaum and Sahl, 1987).

The most conspicuous phenotypic difference between wild type and LTA mutant was observed when physicochemical properties of the bacterial cells were compared. The SA113 *ypfP* mutant had completely lost its ability to form a biofilm on hydrophobic polystyrene plates, which is most probably due to its inability to adhere to the surface. Accordingly, the mutant exhibited a significant change in surface hydrophobicity. These data demonstrate a critical role of LTA in shaping the physicochemical surface properties of *S. aureus*. As biofilm formation on artificial hydrophobic implants such as catheters, joints, heart valves or pace makers represents a major cause of staphylococcal infections (Davey and O'Toole, 2000; Götz, 2002), our data put LTA and YpfP into the position of very interesting targets for novel antibiofilm agents.

Taken together, our study indicates that LTA and LTA-biosynthetic enzymes may represent important targets for novel anti-infective therapies. A *Streptococcus agalactiae* mutant with altered LTA glycolipid structure has recently been shown to have a defect in interactions with brain endothelial cells (Doran *et al*., 2005) indicating that LTA may have additional adhesive functions during infections. Elucidating the relevant, probably multiple functions of LTA and related lipoglycans in Gram-positive cell envelopes and characterizing the major enzymes of the biosynthetic pathways represent important aims for future research.

## Experimental procedures

### Bacterial strains and growth conditions

*Staphylococcus aureus* SA113, a derivative of *S. aureus* 8325 (Iordanescu and Surdeanu, 1976), is a frequently used laboratory strain (Cramton *et al*., 1999; Peschel *et al*., 1999; Weidenmaier *et al*., 2004). Unless otherwise noted, bacteria were grown in BM broth (1% tryptone, 0.5% yeast extract, 0.5% NaCl, 0.1% K_2_HPO_4_, 0.1% glucose) supplemented with appropriate antibiotics. In order to compare growth kinetics, overnight cultures of the test strains were diluted to an optical density (OD) of 0.05 at 578 nm and incubated with shaking at 37°C while the turbidity was regularly monitored. Bacterial viability under starvation was determined by continued shaking at 37°C of late-stationary-phase cultures (after cultivation for 24 h) and counting colony-forming units (cfu) after plating appropriate dilutions of consecutive samples on BM agar plates. To analyse halotolerance, BM overnight cultures were used to inoculate BM containing 2.5 M NaCl and aerobic growth at 37°C was monitored for 30 h. MIC values were determined by diluting bacteria from overnight cultures to an OD_600_ of 0.1 in fresh LB-Lennox medium containing serial dilutions of antimicrobial peptides. OD at 600 nm was monitored after 6 h of aerobic growth at 37°C. Susceptibility to vancomycin was determined by *E*-test as described by the manufacturer (Inverness Medical Deutschland GmbH).

### Construction and plasmid complementation of *ypfP* mutants

The *ypfP* gene of *S. aureus* SA113 (codon 37–333 out of 409) was replaced by an erythromycin resistance cassette (*ermB*) derived from pEC1 or by a spectinomycin adenyltransferase gene (*spc*) from Tn*554* (Murphy *et al*., 1985). Upstream (1.19 kb) and downstream (0.93 kb) flanking regions of the *ypfP* gene were amplified by polymerase chain reaction (PCR) and inserted into pBT2 (Brückner, 1997) along with the 1.5 kb *ermB* fragment or inserted into pMAD (Arnaud *et al*., 2004) along with the 1 kb *spc* fragment using appropriate restriction enzymes. *ermB* or *spc* contained no transcriptional terminators thereby minimizing the risk of polar effects. The resulting plasmids were transferred into SA113 and gene replacement was allowed to take place by incubating the temperature-sensitive plasmids at 42°C according to standard procedures (Brückner, 1997; Arnaud *et al*., 2004). The proper integration of *ermB* and *spc* was verified by PCR.

To complement the SA113 *ypfP* mutant, plasmid pRB473ypfP containing the *ypfP* gene with its putative promoter (440 bp upstream of the translational start) was constructed by PCR-amplifying the region of interest and subsequent cloning into the shuttle vector pRB473 (Brückner, 1992) using appropriate restriction enzymes.

All PCR-amplified DNA fragments were verified by sequencing. *S. aureus* transformation was accomplished by electroporation (Augustin and Götz, 1990). Other molecular techniques followed established protocols (Sambrook *et al*., 1989; Novick, 1991).

The *ypfP* mutation was transduced from SA113 to RN4220 (and back) by phage transduction using phage Φ11 according to established techniques (Novick, 1991). Successful transfer of the mutation was verified by PCR.

### Glycolipid analyses

Polar lipids of bacteria grown overnight in BM supplemented with 0.15% glucose were extracted by the Bligh–Dyer procedure (Bligh and Dyer, 1959). Lipids, vacuum-dried and dissolved in chloroform/methanol (2:1, v/v), were spotted onto silica 60 F254 HPTLC plates and developed in chloroform/methanol/water (70/30/4, v/v/v). Glycolipids were visualized by spraying with α-naphthol (3.2%) in methanol-H_2_SO_4_-H_2_O (25:3:2, v/v/v) and heating the plate at 110°C for 5–10 min.

To determine the composition of the glycolipid missing in the Δ*ypfP::ermB* mutant, samples of *S. aureus* SA113 were washed from the silica gel with 1 ml of dichloromethane/methanol (2:1). Two aliquots (duplicate analysis) of 250 μl were taken, arabinose (26.3 μg) added as internal standard and the solutions dried under vacuum. One hundredmicrolitres of 0.6 N HCl in methanol and 10 μl methyl acetate were added and held at 70°C for 20 h. Ten micrograms of n-tetracosane was added as second internal standard, the methanolic solution was cooled to 0°C and extracted twice with n-heptane (200 μl and 100 μl respectively). The heptane fractions were concentrated to 20 μl and analysed by GC via flame ionization detection on a DB 5 capillary. After addition of 10 μl of tert-butanol, the methanol fractions were taken to dryness under vacuum, 50 μl of bis(trimethylsilyl)trifluoroacetamide/acetonitrile (1:1) was added and held at 60°C for 30 min. The reaction mixture was analysed by GC-MS on a DB 5 capillary.

### Quantification of LTA by ELISA

In order to determine the amount of cell-associated LTA, bacteria grown to logarithmic phase or overnight in BM supplemented with 0.15% glucose were washed twice with sodium citrate buffer (100 mM, pH 4.7), and disrupted mechanically with glass beads (Sigma) in a FastPrep® Instrument (Qbiogene). Cell lysates were extracted for 30 min at room temperature with an equal volume of n-butanol to remove phospholipids and other amphiphilic substances. The butanol phases of wild-type and *ypfP* mutant lysates contained no detectable amounts of LTA in pilot experiments and were not further used for LTA quantification. The LTA-containing aqueous phases were dried under vacuum and re-suspended in the same volume of ultrapure water as before. These preparations or culture supernatants were appropriately diluted in sodium citrate buffer and incubated overnight at room temperature in polystyrene 96-well plates (flat bottom, high binding, type I, Corning). LTA was quantified as described previously (van Langevelde *et al*., 1998). Briefly, monoclonal anti-LTA antibody, clone 55 (mouse IgG_3_; > 0.2 mg ml^−1^; Dunn Labortechnik GmbH, Asbach, Germany), diluted 1:500 was incubated in LTA-coated microtitre plates for 1 h followed by incubation with a 1:5000 dilution of goat anti-mouse IgG peroxidase conjugate (Calbiochem) for 1 h both at room temperature. One hundred microlitres of substrate solution (Substrate Reagent Pack, R&D Systems) was subsequently added, reaction was stopped after 20 min by addition of 50 μl H_2_SO_4_ (1 M) and absorption at 450 nm was determined. The LTA concentration was calculated using *S. aureus* standard LTA (Sigma) appropriately diluted (0–500 ng ml^−1^). To determine the LTA content in the supernatant of SA113 Δ*ypfP::ermB* (pRB473ypfP), bacteria were cultivated without chloramphenicol, which is known to inhibit the release of LTA (Pollack *et al*., 1992).

In order to verify that the ELISA detects LTA from wild-type and *ypfP* mutant strains with equal efficiency we used purified SA113 LTA [pools that eluted between 20% and 40% 1-propanol in ammonium acetate buffer (400 mM, pH 4.7) from an octylsepharose column] from both strains. These elution fractions revealed similar amounts of LTA per mol phosphate (wild type: 529 μg LTA μmol^−1^ phosphate, *ypfP* mutant: 576 μg LTA μmol^−1^ phosphate). Organic phosphate was determined by a colorimetric assay according to Chen *et al*. (1956).

### Quantification of WTA

Wall TA was quantified according to established procedures (Jenni and Berger-Bächi, 1998; Weidenmaier *et al*., 2004). Bacteria were grown overnight in BM containing 0.25% glucose, washed twice in ammonium acetate buffer (20 mM, pH 4.7) and disrupted in the same buffer (2 ml per gram wet weight) by vortexing with glass beads (4.5 g per gram wet weight) for 5 min (three cycles). In between samples were rested on ice. This disruption procedure was repeated once. Crude cell lysates were pooled and incubated overnight with DNase I (Roche; 40 units ml^−1^) and RNase A (Sigma; 80 units ml^−1^) at 37°C. Subsequently, SDS was added (final concentration 2%) and cell extracts were vigorously shaken for 1 h at 65°C. Cell walls were sedimented by centrifugation and subjected to repeated washings with ammonium acetate buffer to remove SDS and soluble cell components. WTA was then released from peptidoglycan by diluting samples fourfold in 5% trichloroacetic acid in ammonium acetate buffer and incubating for 4 h at 65°C. Cell walls were removed by centrifugation. WTA was quantified by determining its phosphate content as described above. Residual amounts of contaminating phosphate, determined in corresponding samples of a WTA-deficient *S. aureus* SA113 *tagO* mutant (Weidenmaier *et al*., 2004), were substracted.

### Chromatographic purification of LTA

Purification of LTA was performed as described previously (Morath *et al*., 2001) with some modifications. Briefly, bacteria grown overnight in BM supplemented with 0.15% glucose were washed twice with sodium citrate buffer (100 mM, pH 4.7), and disrupted in the same buffer with glass beads in a cell mill (Bernd Euler Biotechnologie Mikrobiologie, Frankfurt, Germany). Cell lysates were extracted for 30 min at room temperature with an equal volume of n-butanol. The aqueous phase was lyophilized and re-suspended in the same volume of ultrapure water as before. The aqueous phase was digested with DNase I (50 units ml^−1^) and RNase A (50 units ml^−1^) for 24 h at 37°C and subsequently 24 h at 50°C with proteinase K (Fermentas Life Sciences, 150 μg ml^−1^). After centrifugation for 15 min at 45 000 *g* the supernatant was subjected to HIC on octylsepharose (Amersham). Linear gradient elution from 16% to 60% 1-propanol in ammonium acetate buffer (100 mM, pH 4.7) was performed. The vast majority of LTA eluted at a concentration of approximately 30–36% 1-propanol.

### Structural analysis of the LTA lipid anchor

For compositional analyses, purified LTA was methanolysed with 0.5 M HCl in methanol at 85°C for 45 min. After removal of the solvent, the products were peracetylated with Ac_2_O in pyridine (1:1.5, v/v, 85°C, 20 min). For fatty acid analysis, LTA was methanolysed with 2 M HCl in methanol at 85°C for 20 h. The sugar and fatty acid derivatives were analysed by GLC on a Hewlett-Packard HP 5890 Series II chromatograph, equipped with a 30 m fused-silica SPB-5 column (Supelco) using a temperature gradient of 120°C (3 min) → 320°C at 5°C min^−1^, and GLC-MS on a Hewlett-Packard HP 5975 inert XL mass selective detector equipped with a 30 m HP-5MS column (Hewlett-Packard) under the same conditions as for GLC. CI was carried out with ammonia as reacting gas.

For the analysis of the glyceryl β-gentiobioside LTA lipid anchor, both LTA preparations from SA113 wild type (5 mg) and from the *ypfP* mutant (3 mg) isolated by HIC purification were treated with 250 μl of aqueous HF (48%, w/v) at 4°C for 24 h in order to cleave the [Gro-*P*]_n_ repeating units. Residual HF was evaporated and the product was subjected to mild alkaline methanolysis (0.1 M NaOMe, 37°C, overnight) in order to selectively remove fatty acid residues from the LTA lipid anchor. The water-soluble glycan backbone was further purified by GPC on a Sephadex G-10 column (2.5 × 110 cm) in 20 mM NH_4_HCO_3_ as solvent monitoring the eluate by a refractometer (Knauer 1000). The purified glycerol β-gentiobioside (yield 1.2 mg for the wild type) was analysed by one-dimensional (1D) and 2D homonuclear ^1^H NMR spectroscopy and, after per-O-acetylation, by GLC-MS as described above for compositional analysis.

### NMR spectroscopy

Prior to measurements, the samples were lyophilized twice from ^2^H_2_O (Eurisotope, D 99.8%). The ^1^H NMR spectra were recorded with a DRX-600 spectrometer (Avance, Bruker, Germany) at 600 MHz, at 300 K in ^2^H_2_O (Eurisotope, D 99.96%). Chemical shifts were referenced to internal sodium 3-trimethylsilylpropanoate-*d*_4_ (δ_H_ = 0). Bruker standard software xwinnmr 3.1 was used to acquire and process all 1D and 2D data (TOCSY and COSY respectively).

### Isolation and detection of surface proteins

Surface proteins were isolated from cells grown to logarithmic or stationary phase at 37°C. Cells washed twice with Tris/HCl buffer (20 mM, pH 8.0) were re-suspended in Tris/HCl buffer (20 mM, pH 8.0) containing 20% d-sucrose (1 ml per mg wet weight) and incubated for 5 min at 37°C with lysostaphin (Sigma, 50 μg ml^−1^). Released surface proteins were collected after centrifugation (2500 *g*, 30 min, 4°C) and quantified using the Quick Start Protein Assay kit (Bio-Rad Laboratories GmbH, Munich, Germany). SA113 wild-type and *ypfP::ermB* mutant strains did not differ in overall amounts of cell wall proteins when normalized for OD. Proteins were separated by SDS-PAGE on Tris/glycine gels and subsequently stained with Coomassie brilliant blue R-250.

For the detection of murein hydrolases, zymographic analyses were performed as described previously (Sugai *et al*., 1990). In brief, surface proteins (20 μg) of cells grown to logarithmic phase were separated on a 10% SDS-polyacrylamide gel containing a final OD_578_ of 22 of heat-killed *Micrococcus luteus*, *S. aureus* SA113 wild-type or SA113 Δ*ypfP::ermB* mutant cells. After electrophoresis, gels were washed for at least 60 min in deionized water to remove SDS and incubated overnight at 37°C in renaturing buffer (100 mM sodium phosphate, pH 7.0). Clear zones of hydrolysis were observed in front of a dark background.

### Autolysis assay

The water-induced autolysis assay was performed as described previously (Peschel *et al*., 2000) with some modifications. Briefly, cells grown to mid-exponential phase were washed twice with sodium phosphate buffer (10 mM, pH 7.0) and autolysis was induced by washing the cells with ice-cold ultrapure water. Cells were re-suspended in sodium phosphate buffer (10 mM, pH 7.0) containing Triton X-100 (0.05%) and autolysis was monitored at 30°C for 2.5 h by determining the decrease in A_600_.

### Microbial affinity for organic solvents

The hydrophobicity of microbial cells was compared by analysing their distribution in hydrophobic and hydrophilic solvents as described by Reid *et al*. (1992), except that dodecane was used instead of hexadecane. Briefly, cells were grown to logarithmic phase, washed twice in sodium phosphate buffer (10 mM, pH 7) and re-suspended in the same buffer to give an A_600_ of 0.44–0.62 (A_0_). Next, 150 μl of dodecane was added to 3 ml of bacterial suspension and the two-phase system was vortexed for 1 min. After a 10 min period of phase separation, the absorption of the aqueous phase was measured again (A). The percentage of cells in the dodecane fraction as a measure of hydrophobicity was calculated by the formula: per cent hydrophobicity = [1 –(A/A_0_)] × 100.

### Biofilm formation

Biofilm assays were performed as described previously (Heilmann *et al*., 1996b), except that 96-well flat bottom polystyrene microtitre plates (Greiner Labortechnik, Frickenhausen, Germany) were used instead of U-bottom. *S. aureus* strains grown aerobically overnight at 37°C in tryptic soy broth (TSB, Oxoid) supplemented with 0.25% glucose were used to inoculate cultures grown in the same medium for 24 h at 37°C without agitation. Supernatants were discarded and remaining surface-absorbed cells in the glass tubes were stained with safranin.

## Supplementary material

The following supplementary material is available for this article:

Table S1 ^1^H NMR (600 MHz) data of glycerol gentiobiose pseudotrisaccharide {3-O-[6-O-(- b-D-glucopyranosyl)-b-Dglucopyranosyl]- sn-glycerol} isolated from the lipid anchor of LTA in S. aureus wild type (D2O, 300 K; internal TSP, dH = 0.00).

This material is available as part of the online article from: http://www.blackwell-synergy.com/doi/abs/10.1111/j.1742-4658.2006.05854.x (This link will take you to the article abstract).
