# Supplementary material for: A Staphylococcus aureus ypfP mutant with strongly reduced lipoteichoic acid (LTA) content: LTA governs bacterial surface properties and autolysin activity
Source: Mol Microbiol. 2007 Aug;65(4):1078–91. doi: 10.1111/j.1365-2958.2007.05854.x (PMC2169524; doi:10.1111/j.1365-2958.2007.05854.x)
Supplement: Table S1 — 1H NMR (600 MHz) data of glycerol gentiobiose pseudotrisaccharide {3-O-[6-O-(- b-D-glucopyranosyl)-b-Dglucopyranosyl]- sn-glycerol} isolated from the lipid anchor of LTA in S. aureus wild type (D2O, 300 K; internal TSP, dH = 0.00). [file mmi0065-1078-SD1.pdf]

Table S1. 600 MHz ^{1}H-NMR data of glycerol gentiobiose pseudotrisaccharide {3-O-[6-O-(-β-D-glucopyranosyl)-β-D-glucopyranosyl]-sn-glycerol} isolated from the lipid anchor of LTA in *S. aureus* wild-type (D_2O, 300 K; internal TSP, δ_H = 0.00).

Residue	^{1}H-NMR data			(Thompson *et al.*, 2002)
	Chemical shift	Coupling constant		Chemical shift of gentiobiose β-D-Glc*p* II-(1→6)-β-D-Glc*p* I
	δ (*ppm*)	*J* (Hz)		δ (*ppm*)
**β-D-Glc*p* II-(1→**				
H-1	4.48	$J_{1,2}$	7.9	4.52
H-2	3.31	$J_{2,3}$	9.4	3.33
H-3	3.48	$J_{3,4}$	9.4	3.51
H-4	3.37	$J_{4,5}$	9.4	3.40
H-5	3.48	$J_{5,6a}$	n.d.	3.47
H-6a	3.70	$J_{6a,6b}$	n.d.	3.73
H-6b	3.93	$J_{6b,5}$	n.d.	3.93
**→6)-β-D-Glc*p* I-(1→**				
H-1	4.45	$J_{1,2}$	8.1	4.66
H-2	3.29	$J_{2,3}$	9.4	3.26
H-3	3.58	$J_{3,4}$	9.4	3.48
H-4	3.59	$J_{4,5}$	9.4	3.47
H-5	3.60	$J_{5,6a}$	n.d.	3.63
H-6a	3.83	$J_{6a,6b}$	11.4	3.85
H-6b	4.19	$J_{6b,5}$	2.0	4.21
**→3)-Gro***				
H-1a	3.67	$J_{1a,1b}$	n.d.*	
H-1b	3.88	$J_{1b,2}$	n.d.*	
H-2	4.15	$J_{2,3a}$	n.d.*	
H-3a	3.76	$J_{3a,3b}$	n.d.*	
H-3b	3.88	$J_{3b, 2a}$	n.d.*	

* Signals for Gro were badly resolved due to overlapping not completely degraded [Gro-*P*]$_{1-3}$ fragments. n.d., not determined.
